# Groundwater depletion in India: the influence of seasonal precipitation and land use land cover

**DOI:** 10.1007/s11356-025-37274-7

**Published:** 2025-12-11

**Authors:** Yanping Cao, Shouraseni Sen Roy

**Affiliations:** 1 College of Geographical Sciences, Faculty of Geographical Science and Engineering, Henan University, Zhengzhou, 450046 China; 2 Key Laboratory of Geospatial Technology for the Middle and Lower Yellow River Regions, Ministry of Education, Henan University, Kaifeng, 475004 China; 3 Department of Geography & Sustainable Development, University of Miami, Coral Gables, FL USA

**Keywords:** Groundwater, India, GRACE, Seasonal Precipitation, Land use land cover change, Statistical methods, Linear Regression, Correlation

## Abstract

The rapid depletion of groundwater across India has been revealed through data from the Gravity Recovery and Climate Experiment (GRACE) satellites, particularly in the northern plains. In India, groundwater supports more than 60% of the irrigated area. The impact of seasonal precipitation and land use land cover change (LULCC) on groundwater was investigated through a correlation analysis of multi-source datasets. The data comprised of regional-level monthly precipitation data from ECMWF Reanalysis v5 (ERA5), LULCC data from the NASA MODIS satellite sensor, and groundwater storage estimates derived from the GRACE satellites’ Center for Space Research (CSR) RL06 Mascon solutions alongside the global land data assimilation system (GLDAS) products. Analysis revealed substantial variability in seasonal precipitation, with the steepest declines observed in northeastern India. Significant expansions in land use types were found for croplands (9%), deciduous broadleaf forests (53%), mixed forests (21%), and urban areas (5%). In northern regions, a negative correlation between groundwater levels and both urbanization and cropland expansion was observed. Moreover, the relationship between seasonal precipitation and groundwater storage demonstrated marked regional and temporal differences. These findings underscore the necessity for integrated water management strategies, including optimizing and diversifying water sources for agriculture, promoting aquifer recharge, and enhancing wastewater treatment practices.

## Introduction

Groundwater constitutes approximately 99% of Earth's liquid freshwater resources (Lall et al. 2020) and serves as the primary source of freshwater in numerous regions globally. Population growth and climate-induced extremes—especially extended drought periods—have intensified reliance on groundwater for domestic, agricultural, and industrial activities. This rising dependence has led to a marked acceleration in global groundwater depletion rates, which have doubled in recent decades due to unsustainable extraction practices driven by increasing demand (Konikow 2011; Wada et al. 2011).

The launch of the Gravity Recovery and Climate Experiment (GRACE) mission in 2002 greatly advanced global hydrological monitoring, offering unparalleled insights into terrestrial water storage dynamics in previously understudied regions. GRACE satellite gravimetry data have highlighted significant patterns of groundwater depletion, with 21 out of the world's 37 largest aquifers exhibiting unsustainable withdrawal rates (Richey et al. 2015). The situation is particularly acute in the Indian subcontinent, where the Gangetic Basin—a key resource for one of the world's most densely populated agricultural regions—shows some of the highest rates of depletion globally with marked seasonal fluctuations (Tiwari et al. 2009; Cao and Sen Roy 2018).

At all India level, groundwater plays an essential role, fulfilling between 50 and 80% of domestic water usage and supporting more than 60% of irrigation needs (Gandhi and Bhamoriya 2011; Panda 2011; Saha et al. 2017). Increasing dependence on groundwater has resulted in widespread depletion, influenced by factors such as rapid population expansion, reduction in agricultural landholding sizes, and Intensification of land-use practices. Together, these elements have pushed many aquifers beyond sustainable limits. Notably, agriculture accounts for approximately 90% of India’s annual groundwater usage (Pati 2021), emphasizing the urgent necessity for enhanced water management strategies to prevent further degradation of this resource.

A significant development occurred in the 1960 s with the implementation of extensive canal irrigation systems across the semi-arid regions of northern and northwestern India. These changes profoundly altered regional hydrological conditions with rapid expansion of agriculture (Badiani-Magnusson and Jessoe 2018). By 2010, almost 39 million hectares of farmland had switched to groundwater irrigation, lowering monsoon-related risks (Siebert et al. 2010). These broad land-use transitions also induced notable regional climate changes, including persistent declines in surface air temperature and increases in average precipitation (Sen Roy et al. 2011).

The ongoing groundwater depletion has led to significant reductions in agricultural productivity, contraction of cultivated land, and diminished yields of critical staple crops such as wheat, rice, and maize (Bhattarai et al. 2021). Rapid urbanization has further aggravated groundwater depletion through greater impervious surface coverage, particularly in metropolitan areas like Delhi and Mumbai, where alterations in land cover have disrupted natural recharge mechanisms (Sen Roy et al. 2020; 2022).

Empirical studies underscore the predominant influence of precipitation on aquifer replenishment, with the summer monsoon playing a disproportionately significant role in groundwater recharge, especially in peninsular India (Asoka et al. 2017; Bhanja et al. 2017; Dangar et al. 2024). While the positive association between precipitation and groundwater recharge is well documented, the spatiotemporal distribution of recharge depends fundamentally on geological context, aquifer hydro-stratigraphy, and site-specific pedological attributes. A noteworthy gap remains in understanding the threefold interactions among seasonal groundwater levels, precipitation variability, and land use/land cover changes within India’s diverse hydrogeological settings. To date, only one comprehensive nationwide study has quantitatively examined this relationship, focusing exclusively on summer monsoon precipitation and corresponding groundwater level fluctuations observed through well networks (Asoka et al. 2018). Importantly, precipitation during non-summer monsoon periods (including winter rains and pre-monsoon showers) may play a substantial role in recharge processes yet remains inadequately explored in current hydrological assessments.

This study provides a systematic evaluation of the effects of regional seasonal precipitation variability and land use land cover change (LULCC) on groundwater dynamics throughout India’s four principal climatic seasons: winter (January to February), summer pre-monsoon (March to May), monsoon (June to September), and post-monsoon (October to December), as classified by the India Meteorological Department (IMD). The comprehensive seasonal framework allows for direct comparison with existing hydrological research, enables a robust assessment of groundwater extraction under varying hydroclimatic conditions, and facilitates analysis from peak recharge intervals to periods of intensive withdrawal.

## Study area

**Fig. 1:**
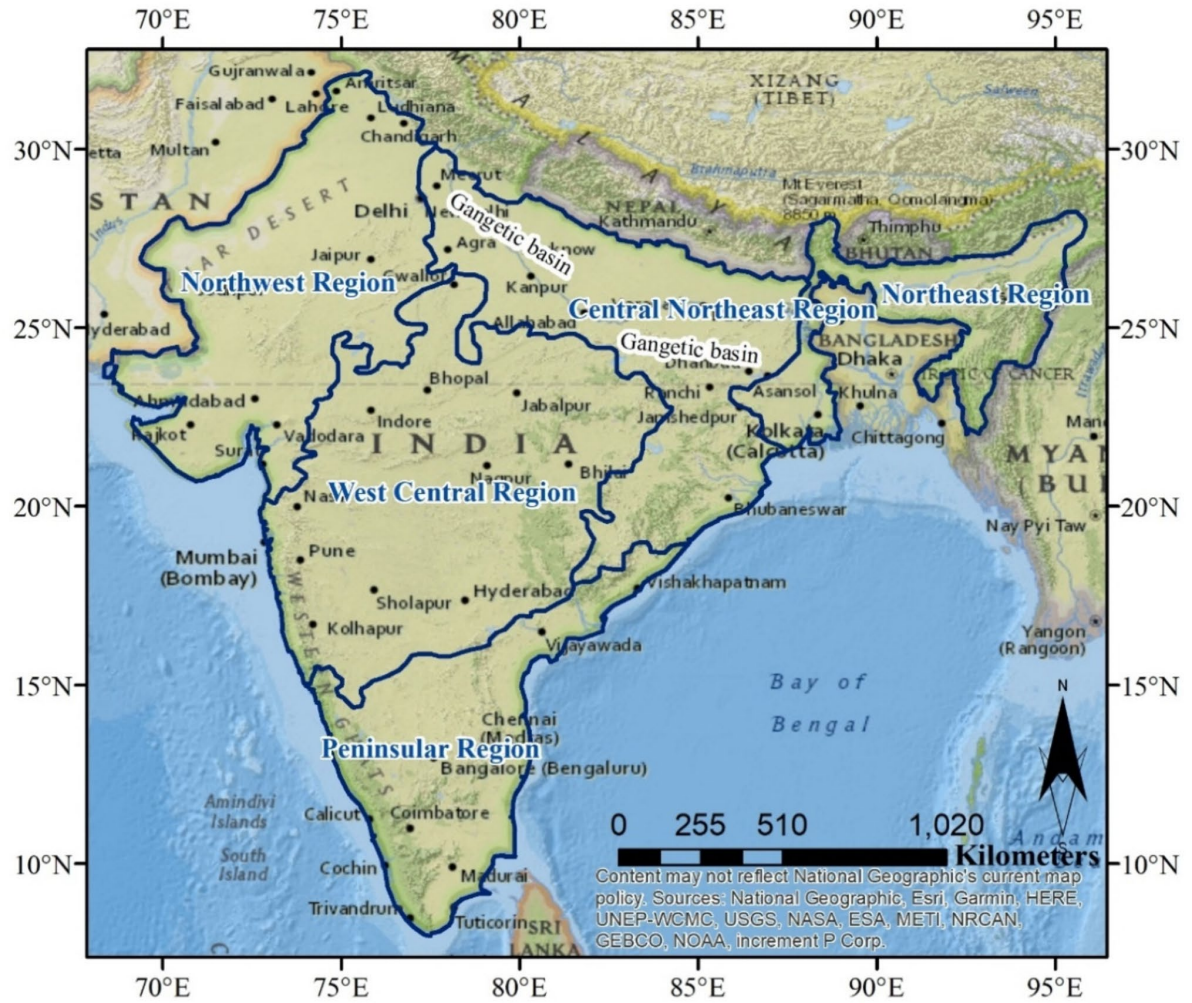
General layout of the study area with demarcation of the five homogenous precipitation regions of India. The northern boundary states of Kashmir and Arunachal Pradesh are not shown on the map because they were not analyzed in this study

India, situated between 8°4'N to 37°4'N latitude and 68°7'E to 97°24'E longitude, has a predominantly tropical climate due to the natural barrier of the Himalayas in the north (Fig. 1). The multi-year average precipitation in the India region is 1206.6 mm, and the multi-year average temperature is 25 °C. This geography leads to significant regional variations in precipitation. The Indo-Gangetic plains are fed by Himalayan glaciers. The Indian subcontinent experiences monsoons, with 70–90% of rainfall occurring from June to September. Precipitation varies spatially, ranging from arid in the northwest to moist tropical in the northeast and west coast. Additional precipitation happens between October and February due to retreating monsoon and western disturbances, mainly affecting the northern plains and east coast (Balachandran et al. 2020).

India faces critical challenges in assessing and developing water resources to meet the needs of its growing population. Precipitation is key for drinking water and agricultural irrigation. Variable precipitation, especially during poor monsoon years, increases groundwater withdrawal, particularly in regions with limited rainfall and frequent monsoon failures (Ramesh and Jagadeeshwari 2012). Groundwater recharge is vital to prevent droughts caused by decreased rainfall, leading to lower water tables and reduced groundwater storage (Pathak and Dodamani 2019). Assessing the impacts of precipitation variability on water resources is crucial given current and projected trends.

## Materials and methods

### Groundwater data

Groundwater storage anomalies were calculated using GRACE satellite data combined with ancillary data from the global land data assimilation system (GLDAS) Noah land surface model. GRACE satellite, a NASA-German Aerospace collaboration, has measured Earth's gravity field since 2002. This study used Center for Space Research (CSR) RL06 mascon solutions (available at http://www2.csr.utexas.edu/grace), recognized for improved accuracy for terrestrial water storage anomalies (TWSA), especially in the Indian subcontinent. TWSA was processed on a 0.25° grid and monthly anomalies (2002–2023) were determined relative to a 2004–2009 baseline. Monthly anomalies for the study period (2002—2023) were obtained by subtracting the climatological monthly average from each corresponding month's value.

To extract groundwater data, GLDAS Noah hydrological variables were utilized via NASA's Goddard Center (https://disc.gsfc.nasa.gov/datasets?keywords=GLDAS), including monthly soil moisture (SM), surface water (SW), and snow water equivalent (SWE). Anomalies for each were computed by subtracting long-term averages, and groundwater anomalies were estimated by subtracting combined SM, SWE, and SW anomalies from TWSA, as expressed in Eq. 1:

1 $$\Delta GW=TWSA-\Delta SM-\Delta SWE-\Delta SW$$

In Eq. (1), $\Delta$ indicates the anomalies, *GW* is groundwater, *SM* is soil moisture, *SWE* is snow water equivalent, and *SW* is surface water. This methodology is widely adopted globally (Solovey et al. 2025), demonstrates its effectiveness in studying groundwater dynamics under diverse hydroclimatic and anthropogenic conditions.

### Precipitation data

To study the correlation between groundwater extraction patterns and climatic variability, we used precipitation data from the European Centre for Medium-Range Weather Forecasts (ECMWF) reanalysis product (ERA5) reanalysis product (Hersbach et al. 2020). ERA5 offers global, high-resolution hourly precipitation estimates from 1940 onward, integrating various observational sources. Research shows that ERA5 captures India's precipitation patterns better than traditional ground-based methods (Bhattacharyya et al. 2022; Mahto and Mishra 2019; Kolluru et al. 2020). For this analysis, we extracted monthly precipitation data from January 2002 to December 2022 to align groundwater storage records and conducted correlation analyses using these datasets.

### Land use land cover change (LULCC) Data

The LULCC data were sourced from NASA's Terra and Aqua satellites via the MODIS Land Cover Type product (MCD12C1, Version 6), which provides annual global land cover classifications at a spatial resolution of 0.05° for the years 2001 to 2022. This dataset is produced using a supervised classification algorithm applied to MODIS surface reflectance, supplemented with prior knowledge and ancillary datasets to achieve higher classification accuracy (Sulla-Menashe and Friedl 2018). The present study specifically examines two predominant land cover categories: croplands and urban areas.

To ensure the reliability of land cover classifications, a rigorous validation process was conducted using high-resolution ground reference data. Furthermore, additional data sets, including regional land use maps and spectral indices—were incorporated to enhance the precision of classification, particularly in distinguishing spectrally similar croplands and peri-urban landscapes. These methodological improvements contributed to more accurate delineation of croplands and urban regions, thereby reducing misclassification errors and strengthening the validity of subsequent analyses.

### Methods

The final analysis was conducted at the regional scale, based on five homogeneous rainfall regions. Linear regression methods (slope) were used to calculate trends in seasonal precipitation and annual LULCC patterns. Hypothesis tests (*p*-value) assessed the significance of these trends at the regional level during the study period.

2 $$slope=\frac{n\times \sum_{i=1}^{n}{x}_{i}\times i-{\sum }_{i=1}^{n}{x}_{i}{\sum }_{i=1}^{n}i}{n\times \left(\sum_{i=1}^{n}{i}^{2}\right)-{\left({\sum }_{i=1}^{n}i\right)}^{2}}$$

where *slope* is the change in the rate of seasonal precipitation and annual LULCC patterns; *n* is the number of years; ${x}_{i}$ refers to the seasonal precipitation or LULCC in year *i*. The change rate is used to quantify the magnitude of the variation in the dependent variable (i.e. the precipitation or LULCC data) with the independent variable (i.e. time), which can reflect trends in precipitation and land cover area.

Furthermore, we calculated the correlation coefficients (*r*) between seasonal-scale groundwater levels, and seasonal rainfall amount, and area under different land cover types, cropland and urban areas, at the regional level. The correlation coefficients were examined by a two-tailed significance test.

3 $$r= \frac{E\left(XY\right)-E\left(X\right)E\left(Y\right)}{\sqrt{{\sigma }^{2}\left(X\right)}\sqrt{{\sigma }^{2}\left(Y\right)}}$$

where, $X$ is groundwater levels and $Y$ is precipitation and LULCC data;$E\left(\right)$ represents the mean and $\sigma \left(\right)$ is the standard deviation. Further information about correlation technique is available from Franzese and Iuliano (2018).

## Results and discussion

### Spatial patterns of trends in seasonal precipitation

**Fig. 2:**
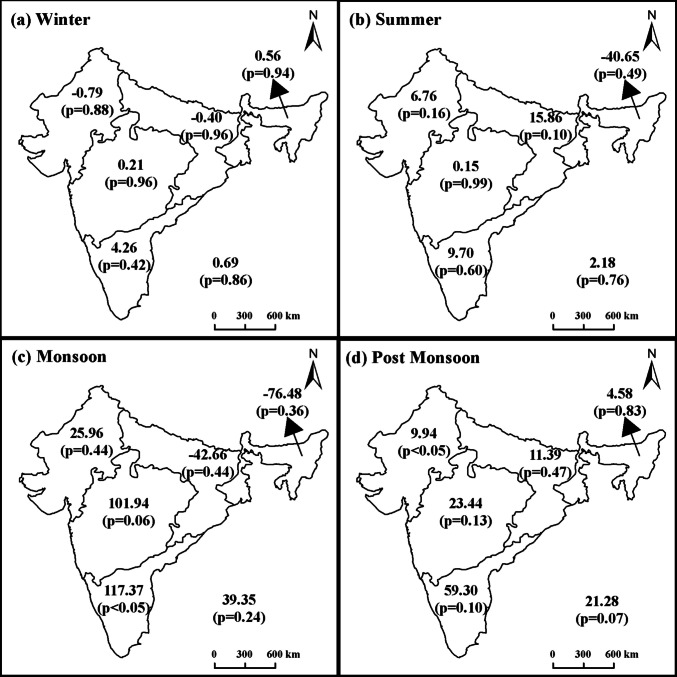
Trends in regional-level precipitation (in 10th of mm/year) (**a**) Winter; (**b**) Summer; (**c**) Monsoon; (**d**) Post Monsoon. Numbers at the lower bottom of each figure is the change slope at all India level. The northern boundary states of Kashmir and Arunachal Pradesh are not shown on the map because they were not analyzed in this study

Regional precipitation trends across India exhibited marked inter-seasonal variability over the study period, as illustrated in Fig. 2. Notably, the northeast region, historically associated with high precipitation, experienced a significant decline during the summer and monsoon seasons, with monsoon precipitation decreasing by −76.48 mm/decade and summer precipitation by −40.65 mm/decade (Fig. 2b, c). In contrast, winter and post-monsoon precipitation in the northeast region showed a modest increase. The northwest region demonstrated a general upward trend in precipitation across most seasons, except for a slight reduction during winter. The central-northeast region presented divergent seasonal patterns, with increases in summer and post-monsoon precipitation counterbalanced by declines in winter and monsoon precipitation. Both west-central and peninsular regions recorded consistent rises in precipitation across all seasons, with the peninsular region experiencing more pronounced increases (Fig. 2). Previously published studies (Das et al. 2014; Dash et al. 2007) have observed similar patterns showing increased rainfall in west-central and peninsular regions, alongside declines in long-term rainfall. These long-term shifts in monsoon precipitation are attributed to changes in cyclonic disturbances and depressions during the monsoon season over the North Indian Ocean. Between 2002 and 2022, all India experienced a consistent increase in seasonal rainfall nationwide. This trend draws attention to the significant variations in precipitation across different regions and times, underscoring the complex climate factors that influence local hydrological patterns.

A distinct east-to-west declining gradient in precipitation was observed across the Indo-Gangetic Basin. This gradient has been partially moderated by the widespread implementation of artificial irrigation infrastructure, particularly canal networks. Extensive irrigation practices, which by the mid-1960s covered over 50% of the basin’s arable land and exceeded 80% in the northwest, have played a pivotal role in shaping local hydroclimatic conditions. Specifically, irrigation increases atmospheric humidity by enhancing evapotranspiration from irrigated surfaces, which can contribute to localized increases in precipitation and modify temperature extremes by reducing daytime highs and increasing nighttime lows (Sen Roy et al. 2011). These irrigation-induced changes are explicitly linked to observed climatic trends, as enhanced atmospheric moisture availability from irrigation has the potential to alter both precipitation and temperature patterns at the regional scale. A recent study by Wang et al. (2024) found that irrigation increased precipitation up to a certain threshold, beyond which the effect reversed.

### Spatiotemporal patterns of LULCC

**Fig. 3:**
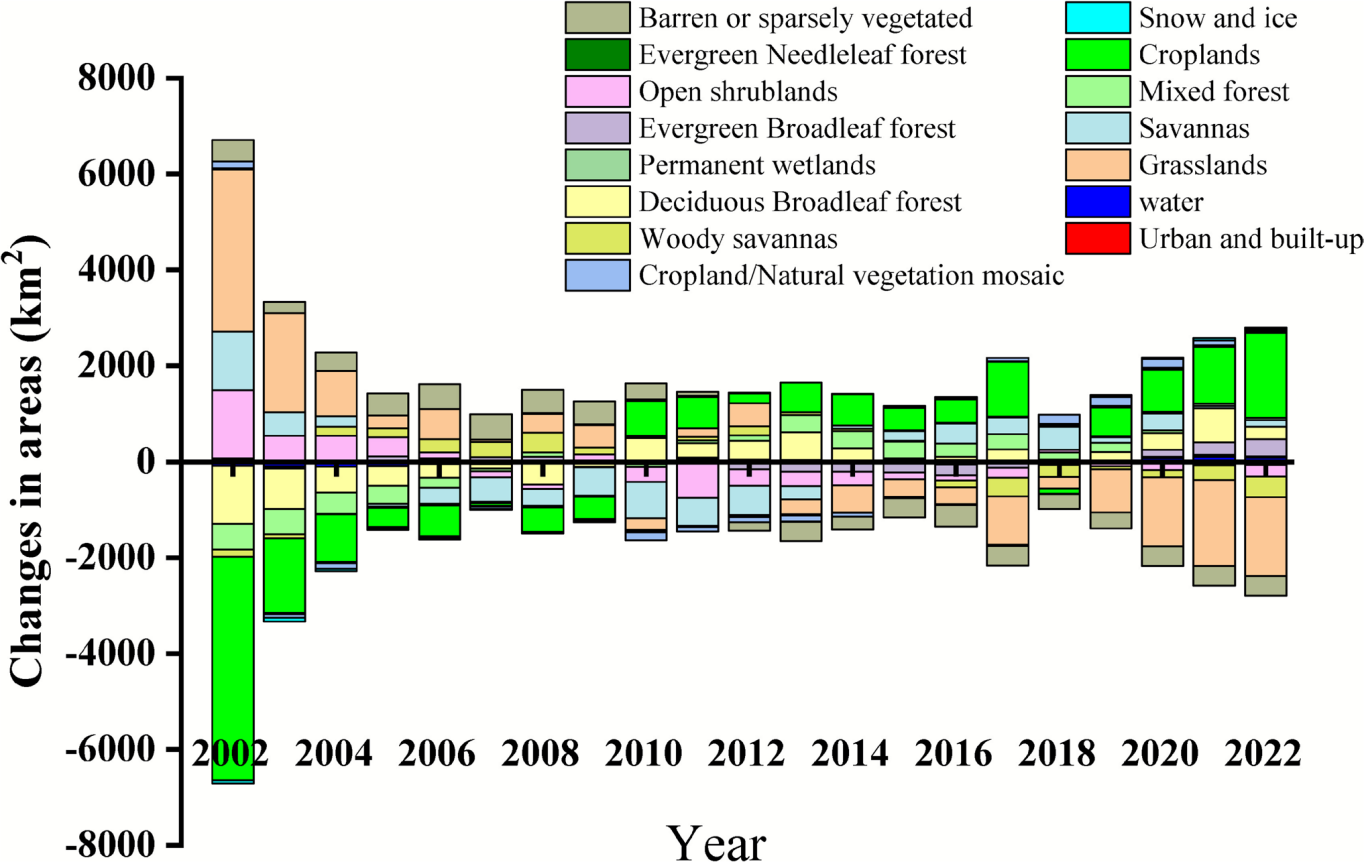
Temporal land use and land cover area anomalies at all India level relative to their respective multi-annual average during 2002 to 2022

Figure 3 shows how land cover and use have changed across India between 2002 and 2022. Over this period, there were significant changes in several key categories: barren and sparsely vegetated regions, croplands, grasslands, savannas, and urban zones. From 2002 to 2011, barren land covered more area than average, but since 2012, it has stayed below average levels. Croplands have steadily increased and remained above average after 2010, apart from a short dip in 2018. Grasslands followed similar patterns to barren lands, with noticeable year-to-year variations. The most dramatic change was in urban areas, which saw rapid and consistent growth throughout the study period. This expansion, paralleling that of croplands, highlights accelerated land conversion driven by both agricultural growth and urban development.

A comprehensive examination of land cover and land use transitions across India offers vital insights into the country’s changing environmental dynamics and related socio-economic developments. The analysis identifies pronounced spatial and temporal variation among six main categories of land cover and use, with significant consequences for sustainable resource management (refer to Table 1).

**Table 1:** Regional level trends in km^2^ for different types of LULCC during 2002–2022

**Regions**	LULCC	Deciduous Broadleaf forest	Mixed forest	Grassland	Cropland	Urban	Barren or sparsely vegetated
**All India**	Trend (km^2^/yr)	1828.51	978.80	−5185.10	5073.61	108.81	−1763.14
	P	*P* < 0.0001	*P* < 0.0001	*P* < 0.0001	*P* < 0.0001	*P* < 0.01	*P* < 0.0001
**Central Northeast**	Trend (km^2^/yr)	425.03	249.58	−320.67	−352.03	31.40	−4.16
	P	*P* < 0.0001	*P* < 0.0001	*P* < 0.0001	*P* < 0.005	*P* < 0.0001	*P* < 0.0001
**Northeast**	Trend (km^2^/yr)	100.69	24.16	54.24	42.76	−0.36	−20.76
	P	*P* = 0.45	*P* = 0.49	*P* < 0.001	*P* = 0.68	*P* = 0.72	*P* < 0.01
**Northwest**	Trend (km^2^/yr)	9.22	4.38	−1173.54	3667.42	26.88	−1468.08
	P	*P* < 0.10	*P* < 0.005	*P* < 0.0001	*P* < 0.0001	*P* < 0.001	*P* < 0.0001
**Peninsular**	Trend (km^2^/yr)	426.75	1.04	−1057.97	580.61	22.92	−3.52
	P	*P* < 0.0001	*P* = 0.98	*P* < 0.0001	*P* < 0.005	*P* < 0.0001	*P* < 0.0001
**West Central**	Trend (km^2^/yr)	832.99	330.43	−2550.93	1940.52	24.56	
	P	*P* < 0.001	*P* < 0.01	*P* < 0.0001	*P* < 0.001	*P* < 0.0001	

At the national scale, croplands displayed the largest expansion (5073.61 km^2^/year, *p* < 0.0001), followed by deciduous broadleaf forests (1828.51 km^2^/year, *p* < 0.0001), mixed forests (978.80 km^2^/year, *p* < 0.0001), and urban areas (108.81 km^2^/year, *p* < 0.01). In contrast, grasslands (−5185.10 km^2^/year, *p* < 0.0001) and barren or sparsely vegetated lands (−1763.14 km^2^/year, *p* < 0.0001) experienced notable reductions.

The most significant land use change occurred in cropland areas, which expanded considerably in most regions. The northwest region demonstrated the highest increase (3667.42 km^2^/year, *p* < 0.0001), followed by the west central region (1940.52 km^2^/year, *p* < 0.001). In contrast, only the central northeast region exhibited a statistically significant decrease (−352.03 km^2^/year, *p* < 0.005). Urban areas generally expanded nearly everywhere, except in the northeast region. Meanwhile, barren and sparsely vegetated lands consistently declined nationwide, reflecting widespread conversion to more intensive land uses.

The northwest and west central regions are characterized by arid to semi-arid climatic conditions, receiving minimal precipitation during the monsoon period. This hydrological limitation has been addressed through the deployment of extensive groundwater-based irrigation systems, enabling substantial agricultural growth. In northwest India, this adaptation has established the area as a leading producer of water-intensive crops such as rice and wheat. LULCC in northwest India has resulted in cooler conditions and lower diurnal temperatures due to increased latent heat flux and reduced sensible heat flux (Prijith et al. 2021). Similarly, the west central region has advanced diversified agriculture, including both staple grains (pulses) and high-value crops (sugarcane, cotton, and oilseeds) (Government of India 2016). These transformations exemplify the intricate interaction between climatic constraints and human-driven innovations within modern agricultural landscapes.

A comprehensive review of existing literature demonstrates that groundwater recharge-depletion dynamics are predominantly influenced by irrigated agricultural practices and urban expansion (Held et al. 2006; Jat et al. 2009; Kumar et al. 2022; Scanlon et al. 2012;). These trends are presenting serious long-term implications for agriculture, ecosystem health, and regional water security. Persistent groundwater extraction threatens agricultural productivity by reducing water availability for crops, undermines ecosystem resilience through diminished base flows and altered habitat conditions, and jeopardizes the sustainability of water resources for future generations. These consequences underscore the urgent need to balance irrigation-driven climatic modifications with responsible water resource management to ensure the long-term viability of agricultural systems, ecosystem integrity, and regional water security. These anthropogenic factors have been shown to significantly alter long-term aquifer sustainability across diverse geographical contexts (Jat et al. 2005; Mapani 2005). Building upon this established scientific consensus, the present study quantitatively examines: (1) the spatiotemporal relationship between seasonal groundwater fluctuations and precipitation patterns, and (2) the association with decadal land-use changes (2002–2022) in cropland coverage and urban development.

### Spatial patterns of seasonal groundwater

**Fig. 4:**
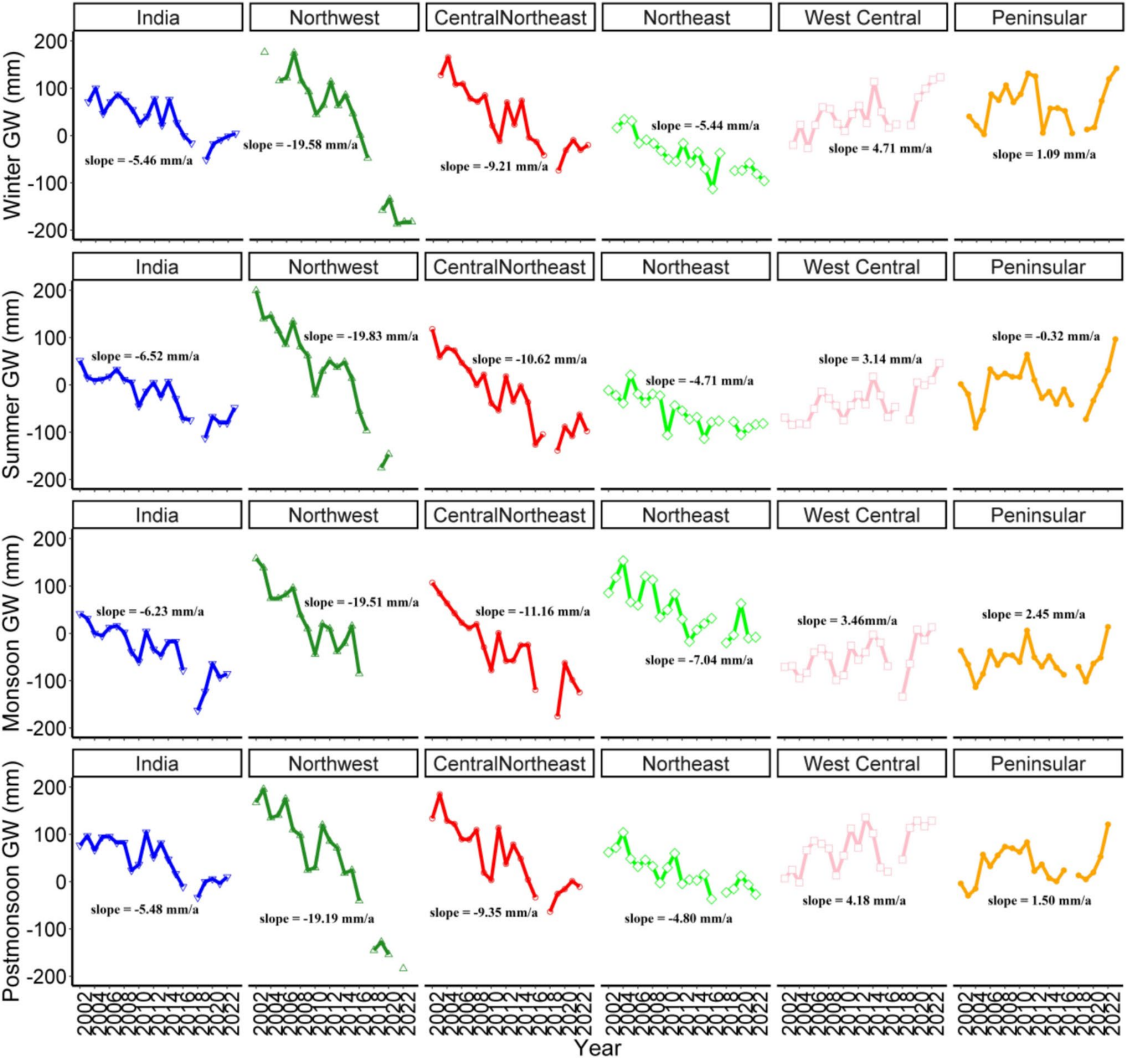
Distribution in regional-level seasonal groundwater (**a**) Winter; (**b**) Summer; (**c**) Monsoon; (**d**) Post Monsoon

Figure 4 shows how groundwater levels across India and its major regions changed seasonally from 2002 to 2022. Across the country, there were significant declines (*p* < 0.001) in groundwater during every season, especially in summer (−6.52 mm/year), while winter saw the least reduction (−5.46 mm/year). Closer examination revealed that the northwest, central northeast, and northeast regions experienced the most severe depletion, with the northwest region suffering the greatest losses. In the northwest region, summer pre-monsoon (−19.83 mm/year, *p* < 0.001) and winter (−19.58 mm/year, *p* < 0.001) declines were about three times higher than the national average.

Additionally, the central northeast region had the largest decrease during the monsoon season (−11.16 mm/year, *p* < 0.001), followed by the northeast (−7.04 mm/year, *p* < 0.001). Conversely, the west central and peninsular areas generally showed improvements, with most seasons seeing an increase in groundwater. The only exception was a minor, statistically insignificant decrease (−0.32 mm/year) during the peninsular summer pre-monsoon period. These trends align with previous hydrological models (Bhanja et al. 2017) and reflect how climate and human activities influence water resources in India's different agro-climatic zones.

Overall, groundwater depletion appeared consistently nationwide, but rates differed greatly between regions (see Fig. 4). The northwest region stood out for its rapid decline throughout all four seasonal cycles (summer, winter, monsoon, and post-monsoon seasons). These trends align with station-level analyses of specific regions in the northwest, which indicate excessive groundwater withdrawal compared to recharge during the monsoon season (Barman et al. 2025). The central northeast region had the next highest depletion, while the peninsular region showed moderate changes. This variation reveals substantial differences in aquifer stress across India’s hydrological areas.

These results highlight the urgent need for science-driven water management in the hardest hit areas. Growing population pressure has led to constant groundwater extraction for both domestic use and irrigation, which limits the replenishment of underground water sources (Barman et al. 2025). The observed patterns of groundwater decline call for swift policy actions, such as introducing integrated water resource management, promoting conservation on the demand side, and advancing climate-resilient farming methods. We also recommend establishing comprehensive, long-term monitoring systems to better understand how climate variability, human activities, and hydrogeological factors interact. Such efforts will be essential for crafting adaptive management strategies that preserve groundwater resources while supporting ongoing socio-economic progress—a vital balance to secure water availability for generations to come.

### Seasonal precipitation and annual LULCC on seasonal groundwater variability

Our multiscale analysis revealed distinct patterns in the relationship between precipitation variability and groundwater storage throughout India. At the national level, groundwater storage consistently showed negative correlations with total precipitation in every season: post-monsoon (−0.34), monsoon (−0.19), summer (−0.19), and winter (−0.03). This pattern suggests that extensive groundwater extraction regularly surpasses the natural recharge processes (see Table 2). However, when examined regionally, these relationships reveal pronounced spatiotemporal differences, reflecting unique hydroclimatic circumstances and varying degrees of anthropogenic pressures. For instance, in the central northeast region, weak positive correlations were observed during the winter and monsoon seasons, while mild negative correlations appeared in the summer and post-monsoon seasons. In the northeast, positive associations during winter, monsoon, and post-monsoon seasons emphasize the dominance of precipitation-driven groundwater recharge. In contrast, the arid northwest displayed strong negative correlations in all seasons except winter, highlighting the region’s heavy reliance on groundwater abstraction for irrigation needs. Collectively, these results indicate that groundwater recharge in India operates over extended timescales, with only the peninsular and west central regions showing seasonally positive relationships between precipitation and storage, likely due to advantageous aquifer characteristics and less intensive extraction.

**Table 2:** Seasonal correlation coefficients between groundwater levels and precipitation, LULCC, from 2002–2022 (The double asterisk (**) indicates 99% level of significance; one asterisk (*) indicates 95% level of significance)

Regions and Factors	Seasons			
	**Winter**	**Summer**	**Monsoon**	**Post Monsoon**
**All India**				
Precipitation	−0.03	−0.19	−0.19	−0.34
Cropland	−0.71**	−0.71**	−0.58**	−0.43
Urban	−0.81**	−0.76**	−0.71**	−0.79**
**Central Northeast**				
Precipitation	0.03	−0.32	0.10	−0.05
Cropland	0.64**	0.57**	0.47*	0.55*
Urban	−0.80**	−0.76**	−0.68**	−0.76**
**Northeast**				
Precipitation	0.42	−0.19	0.40*	0.05
Cropland	−0.61*	−0.35	−0.24	−0.18
Urban	−0.48*	0.31	−0.13	0.02
**Northwest**				
Precipitation	0.04	−0.27	−0.16	−0.61**
Cropland	−0.73**	−0.75**	−0.74**	−0.68**
Urban	−0.85**	−0.65**	−0.68**	−0.75**
**Peninsular**				
Precipitation	−0.05	0.13	0.14	0.15
Cropland	−0.35	−0.33	−0.12	0.28
Urban	−0.01	0.03	0.16	0.16
**West Central**				
Precipitation	0.15	0.26	0.34	0.34
Cropland	0.50*	0.47*	0.34	0.60**
Urban	0.57*	0.50*	0.35	0.38

A review of previous studies further illustrates the complexity of these links. Notably, major rainfall events have been identified as crucial for groundwater recharge in places like the Upper Nile Basin and East Africa (Taylor et al. 2013), a finding that bears relevance for India given the increase in extreme rainfall events brought on by global warming. Precipitation variability is a significant factor explaining changes in groundwater storage in north central and southern India. Nevertheless, factors beyond precipitation, such as LULCC, also exert considerable influence. Negative correlations between groundwater levels and both urban areas and croplands were found in all seasons. Regionally, the northwest and northeast regions showed negative relationships between groundwater and cropland areas, while the central northeast revealed notably negative correlations with urban areas. By contrast, the west central region generally exhibited positive associations with both croplands and urban zones. Supporting this, a case study in Mumbai reported negative links between urban growth and groundwater levels (Sen Roy et al. 2022), underscoring how the expansion of impervious surfaces can restrict recharge. A recent study by Jee et al. (2025) found that groundwater depth is rising due to over-extraction during non-monsoon seasons*.* A similar close relationship between groundwater depth fluctuations and seasonal rainfall patterns was also observed in Southern India (Venkatesan et al. 2021).

In summary, the analysis demonstrates that precipitation alone does not fully explain groundwater storage variability in India. Instead, anthropogenic factors — especially unsustainable groundwater extraction for agriculture and rapid urban development — are decisive contributors. While such practices may support agricultural productivity in the short term, they jeopardize the sustainability of groundwater over the long run. Urbanization compounds the problem by increasing impervious surfaces, which diminish infiltration and increase surface runoff during periods of heavy rain (Miao et al. 2011). Even in the northeast region, where monsoon rainfall is abundant, recharge is limited by both urban expansion and the restrictive nature of mountainous terrain. Major cities like Delhi and Mumbai have experienced steep drops in groundwater levels due to rapid withdrawals and urban sprawl (Sen Roy et al. 2020, 2022). Additionally, rising temperatures have become another key factor, affecting rainfall patterns and driving changes in land cover, thereby heightening the risk of groundwater depletion in other regions (Fatah et al. 2025).

## Implications for groundwater policy in India

Climate change has a pronounced effect on groundwater recharge (Salem et al. 2017). Groundwater is a vital resource in India, especially during the summer monsoon months when rainfall is concentrated. During periods of weak monsoon or drought, groundwater serves as a critical supplement to surface freshwater supplies. Increased groundwater extraction, driven by factors such as population growth, urban expansion, electricity subsidies for agriculture, and climate variability, is exacerbating the crisis. It is estimated that approximately 60% of groundwater sources will reach critical degradation within the next two decades, with northern India enduring the highest levels of depletion (World Bank 2010).

Initiatives to reduce agricultural power consumption and educate farmers on sustainable methodologies have demonstrated success in reversing the decline of groundwater storage in peninsular India (Bhanja et al. 2020; FAO 2022). Groundwater contamination in urban areas presents substantial public health challenges, with unsafe water accounting for 21% of communicable diseases in the country (World Bank 2017). The lack of robust policies regulating groundwater usage stems from limited awareness and insufficiently resourced state agencies. Additionally, disputes over water sharing impede effective regulation and collaborative management (Briscoe 2005).

Unregulated groundwater depletion poses significant risks to food security and public health. Immediate remedial measures include fostering community-based management and implementing location-specific policies adapted to diverse hydrogeological environments. Artificial recharge systems can enhance rainwater supplementation for groundwater replenishment. A holistic strategy is crucial: reducing agricultural demand, addressing supply-side concerns through aquifer restoration, wastewater treatment, and diversification of water resources (Gorton 2017).

## Conclusions

By conducting an extensive assessment of regional hydrologic data collected over two decades (2002–2022), this study systematically investigates the relationship between groundwater behavior and seasonal precipitation, along with land use and land cover changes (LULCC). The results, in alignment with other recent research, reveal considerable declines in groundwater levels throughout the northern plains and northeastern areas of India. The key outcomes of the analysis are outlined below:

- Trends in seasonal precipitation showed substantial fluctuation. The northeastern region underwent the steepest decrease in precipitation, despite typically receiving plentiful annual rainfall. In contrast, both the Peninsular and West Central regions experienced a consistent rise in precipitation across all seasons.
- There was a 9% increase in cropland area, a 53% expansion of deciduous broadleaf forests, a 21% rise in mixed forests, and a 5% growth in urban land. The most pronounced decreases in total area were observed in grasslands and barren lands.
- The relationship between seasonal rainfall and groundwater levels exhibited marked differences across seasons at the regional scale.
- Moreover, the correlation coefficients between groundwater levels and urban development or cropland area were mainly negative (−0.65 to −0.85) throughout northern India. The opposite trend was found across the entire peninsular region.

This study supports prior findings on the interplay among irrigated croplands, urban development, and groundwater levels. It emphasizes the significant reduction in groundwater levels across India (–6.52 mm/year in summer, –5.46 mm/year in winter) and stresses the pressing need for targeted local policy interventions. The ongoing depletion of groundwater threatens food security by lowering crop yields and reducing cropping intensity.

While this study provides important insights into groundwater depletion and its relationship with seasonal precipitation and LULCC across India, several limitations should be acknowledged. First, the reliance on satellite-based datasets (GRACE, MODIS, ERA5, and GLDAS) introduces uncertainties related to spatial and temporal resolution, potential misclassification errors, and limitations in accurately capturing local-scale hydrogeological variations. The GRACE satellite’s coarse spatial resolution may not fully reflect localized groundwater fluctuations, especially in regions with complex aquifer systems or intensive extraction. Additionally, MODIS land cover classifications, though validated, may not distinguish subtle transitions between croplands, peri-urban areas, and other land cover types. The study’s focus on broad regional trends may overlook micro-level influences such as small-scale irrigation practices, local recharge interventions, or land management policies. Furthermore, the correlation analysis used does not establish causality and may be affected by confounding factors such as geological heterogeneity, water table depth, and socio-economic drivers. The exclusion of Kashmir and Arunachal Pradesh from the analysis limits the generalizability of findings to the entire Indian subcontinent. Finally, while the study spans two decades, ongoing climate change and policy shifts may introduce future variability not captured in the present assessment. Future research should aim to integrate higher resolution in situ data, expand geographic coverage, and employ multi-method approaches to enhance understanding of groundwater dynamics amid rapidly evolving environmental and socio-economic contexts. Additional research is needed on the influence of major metropolitan areas on groundwater depletion and the resulting long-term impacts.

## Data Availability

Publicly available.
